# Reproducibility and comparison of a digital food frequency questionnaire (DIGIKOST-FFQ) assessing adherence to national diet and lifestyle recommendations

**DOI:** 10.29219/fnr.v68.10366

**Published:** 2024-08-26

**Authors:** Markus Dines Knudsen, Monica Hauger Carlsen, Anette Hjartåker, Rune Blomhoff, Hege Berg Henriksen

**Affiliations:** 1 Department of Nutrition, Institute of Basic Medical Sciences, University of Oslo, Oslo, Norway; 2 Section for colorectal cancer screening, Cancer Registry of Norway, Oslo, Norway; 3 Department of Transplantation Medicine, Division of Surgery, Inflammatory Diseases and Transplantation, Oslo University Hospital, Oslo, Norway; 4 Department of Clinical Service, Division of Cancer Medicine, Oslo University Hospital, Oslo, Norway

**Keywords:** digital food frequency questionnaire, lifestyle assessment tool, reproducibility, comparison, Norwegian food-based dietary guidelines

## Abstract

**Background:**

We have developed a digital semi-quantitative food frequency and lifestyle questionnaire, the DIGIKOST-FFQ, based on the validated paper-based NORDIET-FFQ.

**Objective:**

The study aims to investigate the reproducibility of the DIGIKOST-FFQ and to compare the DIGIKOST-FFQ against the NORDIET-FFQ for the adjusted questions for intakes of fruits, vegetables, whole grains, fish, meat, and dairy products.

**Design:**

Participants were recruited from May to September 2021 through a random sample from the National Population Register and advertisements on Facebook in Norway. In the reproducibility study, the DIGIKOST-FFQ was completed twice by the participants, 1–2 months apart. In the comparison study, the DIGIKOST-FFQ was completed 1–2 months prior to the NORDIET-FFQ.

**Results:**

In the reproducibility study, 317 individuals were included. For 12 out of 16 food groups there were no significant differences in intake estimations between the first and second DIGIKOST-FFQ administrations. A small but significant median difference was observed for fruits (6 g/day) and vegetables (24 g/day). Correlations were satisfactory for all items (*r* = 0.60–1.00), and in the cross-classification 85% of the participants were classified into the same or adjacent quartile for all items. The comparison study included 81 individuals. Compared to the NORDIET-FFQ a significant median difference was observed for fruits 29 g/day, vegetables 36 g/day, whole grains –10 g/day, and red meat –11 g/day, but not for fish, processed meat, or dairy products.

**Conclusion:**

The DIGIKOST-FFQ was able to reproduce diet and lifestyle at the group level. An intended difference for the food groups where questions had been adjusted, was observed between DIGIKOST-FFQ and NORDIET-FFQ in the comparison study.

## Popular scientific summary

- Digital assessments tools have been shown to be useful when assessing diet and lifestyle and their effects on disease and survival outcomes.
- We investigated the reproducibility of the new digital semi-quantitative food frequency and lifestyle questionnaire, the DIGIKOST-FFQ, and compared the DIGIKOST-FFQ to the paper-based NORDIET-FFQ, which the DIGIKOST-FFQ is based on.
- We observed that DIGIKOST-FFQ was able to reproduce reported dietary intakes, lifestyle- and demographic factors on group levels.
- The adjustments made in the DIGIKOST-FFQ worked as intended.

High-quality assessment tools identifying specific diet- and lifestyle behaviors associated with risk of chronic diseases and other severe health outcomes are of great importance. Digital assessment tools are useful for this purpose (1–3). We have developed a digital diet and lifestyle -questionnaire, the DIGIKOST-FFQ, designed to assess adherence to the Norwegian food-based dietary guidelines (FBDG) and other national lifestyle guidelines (4–6). The DIGIKOST-FFQ is based on the short, paper-based NORDIET-FFQ (7), developed and validated at the Department of Nutrition, University of Oslo (UiO), Norway. However, when developing a new assessment tool it is essential to test the reproducibility to evaluate the precision of estimated intakes and activities by the DIGIKOST-FFQ (8, 9). Additionally, the NORDIET-FFQ was originally used for the dietary assessment in the ongoing CRC-NORDIET study, a comprehensive and long-term intervention and follow-up study of colorectal cancer patients (10). Moreover, the NORDIET-FFQ has been used in other studies such as the ongoing Mom’s Healthy Heart (11) and the VISA study (12, 13). A validation study of the NORDIET-FFQ (7, 14) revealed some challenges and limitations of the tool, that is underestimation of fruit and vegetables, and overestimation of wholegrain. These issues were adjusted for when developing the DIGIKOST-FFQ (6) and it was desired to investigate how the adjustments affected the estimates of the food groups compared to the NORDIET-FFQ. Furthermore, in the CRC-NORDIET study and in the Mom’s Healthy Heart study, the NORDIET-FFQ has and will be replaced by the DIGIKOST-FFQ but in some future studies it may be necessary to use both the paper-based and the digital version in parallel. Therefore, a comparison study is needed to compare estimates of diet and activity estimates between the two assessments.

Thus, in the present study we aimed to investigate the reproducibility of the DIGIKOST-FFQ. Additionally, we investigated how the adjustments from the NORDIET-FFQ to the DIGIKOST-FFQ affected the estimates, by comparing against the NORDIET-FFQ.

## Methods

### Study design and participants

Women and men aged 18 years and older, living in Norway were eligible for both studies. The participants had to be able to read and understand Norwegian and to provide a digital signed informed written consent. In both studies the participants were asked to complete two rounds of questionnaires 1–2 months apart. In the reproducibility study, the participants filled in the DIGIKOST-FFQ twice. In the comparison study, the participants first completed the DIGIKOST-FFQ followed by completion of the NORDIET-FFQ.

The incentive offered to those who completed the studies was an individual report benchmarking dietary intake and physical activity against the Norwegian FBDG. In addition, all participants in each study were enrolled in a lottery of a gift card with a value of NOK 500 (~50 U.S. dollars).

### NORDIET-FFQ

The NORDIET-FFQ has been described in detail previously (7, 14). In brief, the NORDIET-FFQ is a short semi-quantitative paper-based FFQ designed to assess dietary intake (in grams per day) and physical activity, according to the Norwegian FBDG. It includes 63 questions on food items and two questions on physical activity. On average it takes 15 min to complete the NORDIET-FFQ (7).

### DIGIKOST-FFQ

The DIGIKOST-FFQ is an updated digital version of the NORDIET-FFQ. Details about the adjustments have been published earlier (6). In brief, during the fruit intake in the NORDIET-FFQ participants were asked for small-, medium- or large sized fruits, whereas in the DIGIKOST-FFQ they were asked as separate questions on the most commonly eaten fruit species in each category and questions on dried fruit were not included in the DIGIKOST-FFQ. For vegetables, the NORDIET-FFQ included a question on ‘other vegetables’. In DIGIKOST-FFQ, this was changed into separate questions for each item, such as carrots, broccoli, and root vegetables. Furthermore, questions on legumes were added to the DIGIKOST-FFQ. Assistive algorithms were implemented to help in distinguishing between bread with different contents of whole grains, as well as an automatic technical function to calculate number of slices of bread, images of portion sizes, and weight measurements in grams, and new questions on porridge were added to the DIGIKOST-FFQ. Yoghurt was separated as a single category, and images of portion sizes and weight measurements in grams or household measures were added for all dairy products fish and meat products. More details have been published by Henriksen et al. (6).

The DIGIKOST-FFQ includes 103 food and lifestyle questions, out of which 78 are on food items; 7 on physical activity, sedentary time, and sleep; 8 on tobacco use; and 10 questions are about body weight and demographic data. The food groups included in the FFQ cover the most essential food groups in the Norwegian FBDG: foods rich in fiber (i.e. fruit, berries, vegetables, whole grains, and cereals), fish, dairy products, red and white meat, oils, margarine, beverages, and the other lifestyle factors, measured over the last 2 months. It takes approximately 20 min to complete the DIGIKOST-FFQ (4).

### Recruitment of participants

It was estimated that 1,000 and 5,000 individuals should be invited to reach a minimum of 500 and 100 subjects for the reproducibility and the comparison studies, respectively. These numbers of subjects should be adequate to ensure statistical power in the reproducibility and comparison studies (8, 15, 16).

In the reproducibility study, participants were recruited from May to September 2021, and 4,523 individuals were invited by e-mail based on a random selection from the Norwegian National Population Register. Recruitment was also done using Facebook announcements, from which 439 signed up to be invited to the study.

In the comparison study, recruitment took place from May to September 2021 and 862 individuals were invited by e-mail based on a random selection from the Norwegian National Population Register. In addition, participants were invited by a Facebook announcement, from which 111 signed up to be invited to the study.

To be included in either of the two studies, both administrations had to be completed within more than 1 month and less than 2 months apart.

### Ethics

Both studies were carried out in accordance with the Helsinki Declaration. The Data Protection Services (Sikt) has approved the DIGIKOST-protocol and the informed consent (ref. no. 277679). No data were collected about the invitees who did not participate.

### Statistical analysis

Continuous variables are presented as median and inter quartile ranges (IQR). Absolute median differences and IQR were calculated subtracting estimates in the second administration (DIGIKOST-FFQ 2 or the NORDIET-FFQ) from the estimates in the first administration (DIGKOST-FFQ 1 or DIGIKOST-FFQ). To compare differences, paired t-test was used when data was normally distributed and Wilcoxon signed-rank test was used when not normally distributed; correlation was calculated as Pearson’s and Spearman’s correlation coefficients. To evaluate how well the correlations were between the two methods we used the cutoffs defined by Hankin et al., of which a correlation below 0.3 is regarded as poor, between 0.3 and 0.49 as fair, and above 0.5 as satisfactory (17). Categorical variables are presented as frequencies and percentage and interrater agreement by using kappa statistics.

In the reproducibility study, we investigated misclassification by ranking participants intakes into quartiles and investigated how many participants differed with more than +/- one quartile between the two methods (cross-classifications). Bland-Altman plots were used to further explore the differences between methods and reproducibility of the same method (18, 19), such as under- or over -reporting (mean differences), limits of agreement, and presence of outliers in the data.

All analyses were performed using STATA™ software, version 16.0 (Stata Corp, College Station, Texas, USA). *P* < 0.05 was considered statistically significant.

## Results

### Reproducibility study

**Fig. 1:**
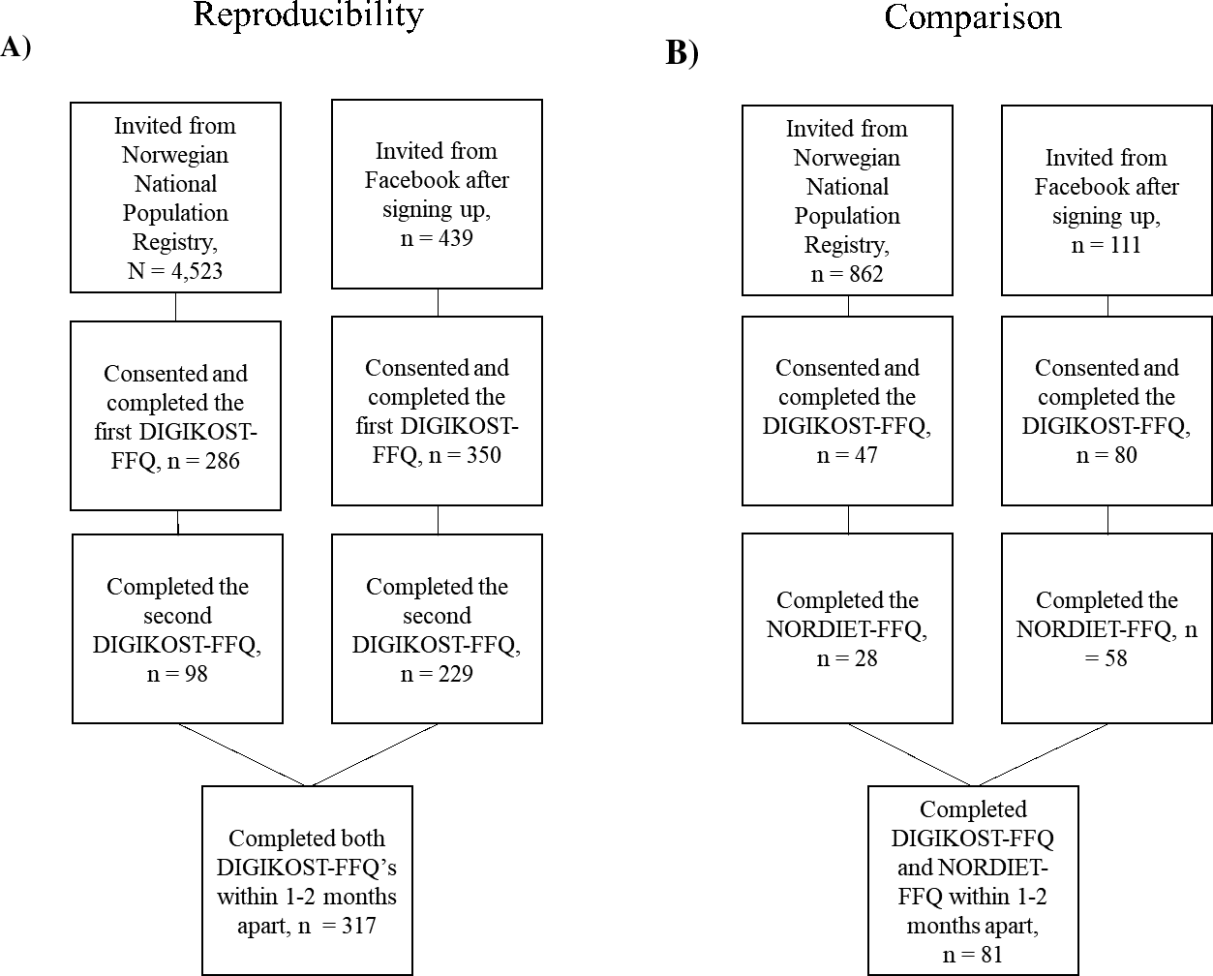
Flowchart of participant selection in (A) Reproducibility study. (B) Comparison study.

A total of 317 answered the DIGIKOST-FFQ twice, corresponding to 6.4% of those invited and 50% of those who consented to participate in the study (Fig. 1A). More than half of the study population had attained education at the university/university college level ≥ 4 years, 85% were women and mean age was 51 years (IQR; 36, 61) (Table 1). Seventy percent of the participants were recruited from Facebook, of which 96% were women, whereas 57% were women among the participants recruited from Norwegian National Population Register (details not shown).

**Table 1:** Reproducibility of demographic factors, *n* = 317

	DIGIKOST-FFQ 1, %	DIGIKOST-FFQ 2, %	Agreement^a^
Women	85		
Ethnicity, mother or father is from Norway	97		
Civil status, cohabitant	77	77	98%
Highest completed educational level			96%
Primary school and lower secondary 7–10 years	1	1	-
Upper secondary school 1–2 years	6	5	-
Secondary school 3 years	4	7	-
University/university college studies of <4 years	26	27	-
University/university college ≥4 years	58	55	-
Certificate of apprenticeship	4	5	-
Working status			94%
Working	64	64	-
Home (self-chosen)	1	1	-
Retired	16	16	-
Unemployed	2	1	-
Sick leave	1	2	-
Rehabilitation	2	2	-
Temporary disability benefits	1	0	-
Permanent disability benefits	9	9	-
Student	6	5	-

a % agreement between DIGIKOST-FFQ 1 and DIGIKOST-FFQ 2, tested by kappa statistics of inter-rater agreement.

### Height, weight, body mass index, physical activity, sedentary time, sleep, alcohol, and tobacco use

**Fig. 2:**
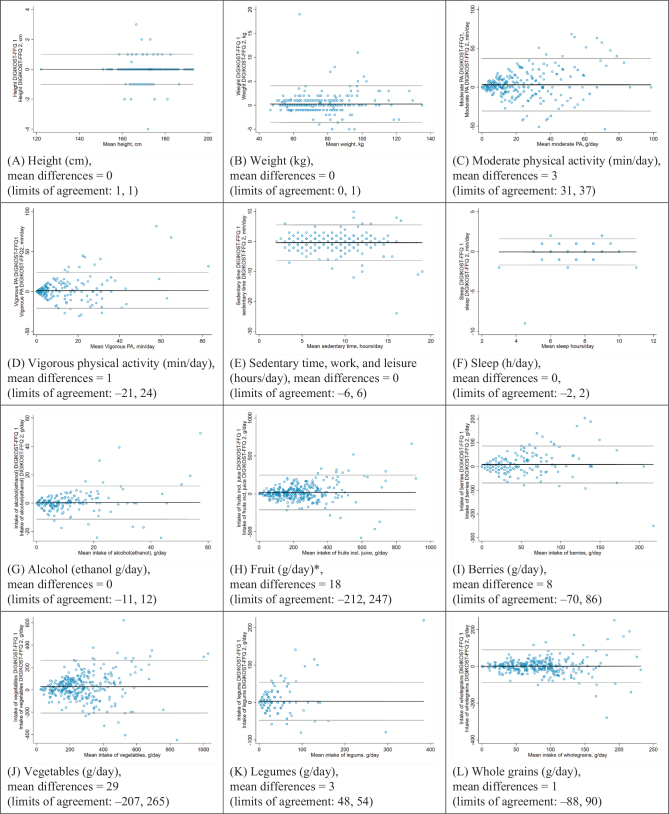

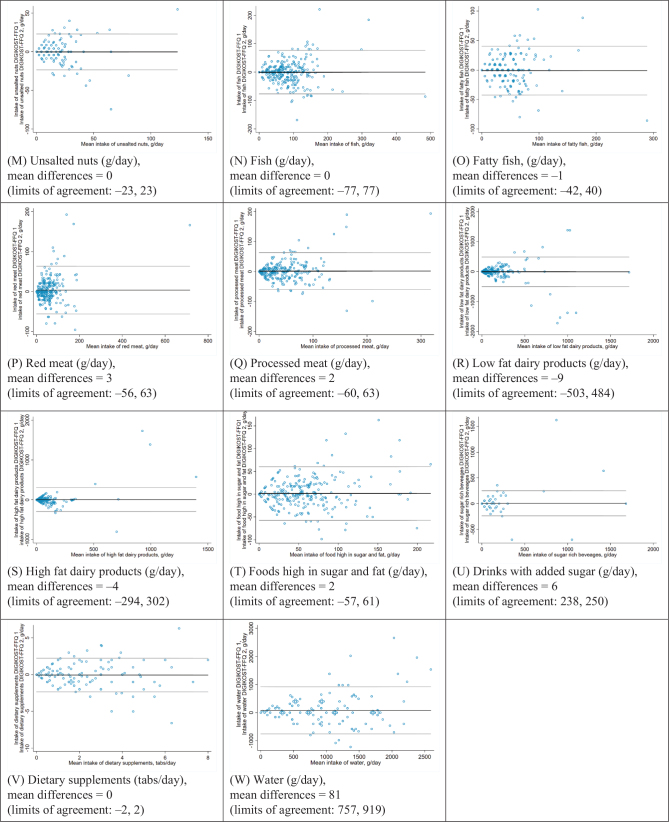
Bland-Altman plots depicting the mean differences in DIGIKOST-FFQ 1 minus DIGIKOST-FFQ 2 for intake of food groups grams per day, moderate and vigorous activity (minutes per day), height (centimeter), weight (kilograms), and sleep and sedentary time (hours per day). The black line represents the mean differences, and the gray lines represent the1.96 SDs limits of agreement, *n* = 317. *incl. berries and juice, maximum one glass of juice per day.

Table 2 shows the results from the reproducibility for ‘height’, ‘weight’, ‘vigorous physical activity’, ‘moderate physical activity’, ‘sedentary time’, ‘sleep’, and intake of ‘alcohol’. Small differences were observed between the first and second administration for these factors. Correlation coefficients varied from 0.63 (sedentary time, work, and leisure) to 0.99 (height and weight). Disagreement was observed for 7% of the participants for ‘moderate physical activity’ and ‘sedentary time’, 12% for ‘vigorous physical activity’, 14% for ‘alcohol’, and 15% for ‘sleep’. Ninety-six percent and 99% of the participants reported as ‘never smoked’ and ‘never used snuff’, respectively, were correctly classified, and none were in disagreement group in the cross-classification analysis. Bland-Altman analyses are presented in Fig. 2A–G. There was a tendency of an increase in differences of reported ‘alcohol’ intake at intakes above 20 g/day between the two completions of the DIGIKOST-FFQ. We also observed a trend of a wider scatter of differences with increasing time in both ‘moderate physical activity’ and ‘vigorous physical activity’. However, for all the factors the majority were within the 95% limits of agreement with an even distribution around the mean.

**Table 2:** Reproducibility of lifestyle factors, *n* = 317

	DIGIKOST-FFQ 1, median (IQR)	DIGIKOST-FFQ 2, median (IQR)	p^a^	Difference, median (IQR)	Correlation^b^	Cross-classification		
						Exact, %	Exact+ adjacent. % ^¤^	Disagreement %^#^
Height, cm ^c^	170 (165, 175)	170 (165, 175)	>0.05	0.0 (0, 0)	0.99*	98	100	0
Weight, kg ^c^	71 (63, 83)	71 (63, 83)	>0.05	0 (0, 0)	0.99*	92	100	0
Vigorous physical activity, min/day^d^	4 (0, 16)	3 (0, 11)	<0.05	0.0 (0, 4)	0.73*	61	88	12
Moderate physical activity, min/day^d^	18 (9, 36)	18 (7, 35)	<0.05	0.6 (–5, 11)	0.72*	56	93	7
Sedentary time, work, and leisure, hours/day ^c^	8 (6, 11)	9 (6, 11)	<0.05	0 (–2, 1)	0.63*	56	93	7
Sleep, hours/day ^d^	7 (7, 8)	7 (7, 8)	>0.05	0.0 (0, 0)	0.77*	79	85	15
Alcohol, g ethanol/day^d^	2 (0, 9)	2 (0, 9)	>0.05	0.0 (0, 1)	0.90*	74	86	14
Categorical variables, %								
Never smoked	53	53	-	-	-	96^	100	0
Never used snuff	90	90	-	-	-	99^	100	0

a *P*-value, test of difference in intake between first and second DIGIKOST-FFQ, by paired t-test when normal distributed and Wilcox.sign. rank test when non-normal distributed. Significant difference set at *P* < 0.05.

b Pearson correlation test for normal distributed variables and Spearman for non-normal distributed variables.

* Significant correlation set at *P* < 0.05.

c Normal distributed, mean and mean differences, and 95% confidence interval. Paired t-test to calculate *P*-value for difference.

d non-normal distributed.

¤ adjacent quartile = ±1 quartile differences between e.g., DIGIKOST-FFQ 1 and DIGIKOST-FFQ 2.

# Disagreement is more than one quartile differences (> ±1 group), e.g. DIGKOST-FFQ 1 = q2, to be in disagreement then DIGIKOST-FFQ 2 = q4.

^ similar as % agreement between DIGIKOST-FFQ 1 and DIGIKOST-FFQ 2, tested by kappa statistics of inter-rater agreement.

IQR, Inter quartile range.

### Fruit, berries, vegetables, legumes, whole grains, and unsalted nuts

Table 3 shows the reproducibility for food intakes. For the fiber rich food groups, the median differences were between 0 and 24 g/day. The intake reported in the food groups ‘fruits’, ‘berries’, and ‘vegetables’, was significantly higher in the first compared to the second administration of the questionnaire. Correlations between the first and second administration of the DIGIKOST-FFQ for these food groups were satisfying; 0.60–0.77 and disagreement ranged from 5 to 11% (Table 3). The Bland-Altman analyses are shown in Fig. 2H–M. Generally, there was a tendency for increasing differences with increasing mean intakes, distributed evenly above and below the mean difference (i.e. both lower and higher intake in the retest compared to the test). Intake of fruit, vegetable, whole grains, and unsalted nuts showed wide limits of agreements; still the limits of agreements were within the medians of the two administrations, except for unsalted nuts (Table 3).

**Table 3:** Reproducibility of dietary factors, *n* = 317

	DIGIKOST-FFQ 1, median (IQR)	DIGIKOST-FFQ 2, median (IQR)	p^a^	Difference, g/day, median (IQR)	Correlation ^b^	Cross-classification, %		
						Exact	Exact+adj.^¤^	Disag^#^
Fruits, g/day* ^d^	220 (126, 305)	198 (121, 291)	<0.05	6 (–29, 64)	0.74*	58	94	6
Berries, g/day ^c^	29 (14, 71)	29 (7, 47)	<0.05	0 (–7, 22)	0.67*	50	89	11
Vegetables, g/day ^d^	283 (176, 360)	258 (147, 350)	<0.05	24 (–28, 83)	0.75*	54	92	8
Legumes, g/day ^c^	14 (0, 29)	7 (0, 29)	>0.05	0 (0, 7)	0.77*	61	91	9
Whole grains, g/day ^c^	81 (45, 103)	80 (48, 105)	>0.05	1 (–16, 18)	0.60*	53	92	8
Unsalted nuts, g/day^c^	9 (0, 21)	9 (0, 21)	>0.05	0 (–3, 2)	0.75*	65	95	5
Total fish, g/day^c^	70 (35, 100)	69 (38, 106)	>0.05	0 (–18, 17)	0.79*	59	93	7
Fatty Fish, g/day^c^	36 (18, 54)	37 (18, 55)	>0.05	0 (–9, 2)	0.82*	59	93	7
Red meat, g/day^c^	44 (21, 57)	39 (21, 67)	>0.05	0 (–11, 14)	0.81*	61	94	6
Processed meat, g/day^c^	27 (8, 54)	27 (8, 57)	>0.05	0 (–8, 11)	0.80*	61	92	8
Low-fat dairy products, g/day^c^	62 (8, 153)	70 (8, 154)	>0.05	0 (–25, 15)	0.74*	62	91	9
High-fat dairy products, g/day^c^	15 (3, 60)	14 (3, 61)	>0.05	0 (–11, 8)	0.66*	59	87	13
Food high in sugar and fat, g/day ^c^	37 (6, 70)	38 (6, 68)	>0.05	0 (–10, 14)	0.81*	61	93	7
Sugar rich beverages, g/day ^c^	0 (0, 0)	0 (0, 0)	>0.05	0 (0, 0)	0.73*	90	90	10
Water, g/day ^c^	1,000 (700, 1,400)	757 (443, 1,243)	<0.05	0 (0, 243)	0.78*	57	93	7
Dietary supplements, tabs/day^c^	1 (0, 2)	1 (0, 2)	>0.05	0 (0, 0)	0.82*	74	92	8

a *P*-value, test of difference in intake between first and second DIGIKOST-FFQ, by paired t-test when normal distributed and Wilcox.sign. rank test when non-normal distributed. Significant difference set at *P* < 0.05.

b Pearson correlation test for normal distributed variables and Spearman for non-normal distributed variables.

* Significant correlation set at *P* < 0.05.

c non-normal distributed.

d Normal distributed, mean and mean differences, and 95% confidence interval. Paired t-test to calculate *P*-value for difference.

¤ Adjacent quartile = ± 1 quartile difference between e.g. DIGKOST-FFQ 1 and DIGKOST-FFQ 2.

# Disagreement is more than one quartile difference (> ±1 group), e.g. DIGKOST-FFQ 1 = q2, to be in disagreement then DIGIKOST-FFQ 2 = q4.

* incl. berries and juice, maximum one glass of juice per day. IQR, Inter quartile range.

### Fish, dairy products, and meat

There were no significant differences between estimates in intakes of ‘fish’, ‘meat’, and ‘dairy products’ between the two administrations of the DIGIKOST-FFQ. The correlation coefficients were satisfactory and ranged from 0.66 to 0.82. The misclassification in quartiles was between 6 and 13% (Table 3). The Bland-Altman analyses are shown in Fig. 2N–S. Some outliers were observed resulting in wide limits of agreements, particularly for ‘low fat dairy products’ and ‘high fat dairy products’. However, for all the factors the majority were within the 95% limits of agreement with an even distribution around the mean.

### Foods high in sugar and fat, sugar rich beverages, water, and dietary supplements

No significant differences were observed between estimated intakes of ‘foods high in sugar and fat’, ‘sugar rich beverages’, or ‘dietary supplements’. The estimated intakes of ‘water’ were significantly different; however the median difference was 0 g/day (IQR; 0, 243). The misclassification for these four food groups ranged from 7 to 10% (Table 3). The Bland-Altman analyses are shown in Fig. 2T–W. There was a tendency for increased differences with increased mean intakes with a few outliers, but with majority being within the 95% limits of agreement. Water showed wide limits of agreement; however, still within the median of the two administrations (Table 3).

Sensitivity analyses were conducted for the correlation for reproducibility stratified by the invitation method for fruit, berries, vegetables, total fish, red meat, processed meat and alcohol, with similar correlation coefficients for Facebook invited and Norwegian National Population Register invited (details not shown).

### Comparison study

A total of 81 participants filled in both the DIGKOST-FFQ and the NORDIET-FFQ, corresponding to 8% of those invited (*n* = 973) and 64% of those who consented (*n* = 127). The flowchart of participants is shown in Fig. 1B. The median age of the participants was 51 years, and 78% were women (Table 4). Most (58%) of the participants were highly educated, 58% had an education at the university/university college level ≥ 4 years (details not shown). Fifty-nine percent of the participants were recruited from Facebook, and 91% of the Facebook recruited were women, whereas among the participants recruited from Norwegian National Population Register 52% were women (details not shown). Diet, lifestyle, and demographics from DIGIKOST-FFQ and NORDIET-FFQ and differences between these are presented in Table 4.

**Table 4:** Comparison of dietary factors measured by the DIGIKOST-FFQ and the NORDIET-FFQ, *n* = 81

Demographic	DIGIKOST-FFQ Median (IQR),	NORDIET-FFQ Median (IQR),	p^a^	Difference, median (IQR)	Correlation^b^
Women %	78				
Age, y	51 (36, 61)	-	-	-	-
**Diet:**					
Fruits, g/day^c^	177 (108, 324)	150 (101, 232)	<0.05	29 (–21, 84)	0.72*
Berries, g/day	29 (14, 47)	16 (8, 31)	<0.05	13 (–3, 28)	0.55*
Vegetables, g/day	226 (138, 373)	173 (98, 295)	<0.05	36 (–10,105)	0.82*
Whole grains, g/day	71 (41, 99)	88 (44, 121)	<0.05	–10 (–36, 10)	0.58*
Total fish, g/day	73 (35, 117)	61 (31, 102)	>0.05	–1 (–20, 24)	0.80*
Fatty fish, g/day	36 (19, 55)	33 (20, 62)	>0.05	–3 (–17, 11)	0.74*
Red meat, g/day	32 (21, 67)	51 (27, 87)	<0.05	–11 (–25, 3)	0.75*
Processed meat, g/day	27 (8, 67)	35 (14, 61)	>0.05	0 (0, 8)	0.75*
Low-fat dairy products, g/day	57 (5, 151)	43 (7, 149)	>0.05	0 (–14, 16)	0.83*
High-fat dairy products, g/day	22 (3, 58)	24 (10, 63)	>0.05	–4 (–19, 8)	0.45*

a *P*-value, test of difference between DIGIKOST-FFQ and NORDIET-FFQ by Wilcox.sign. rank test.

b Spearman correlation test.

* Significant correlation set at *P* < 0.05.

c incl. berries and juice, maximum one glass of juice per day.

IQR, Inter quartile range.

### Fruit, berries, vegetables, and whole grains

**Fig. 3:**
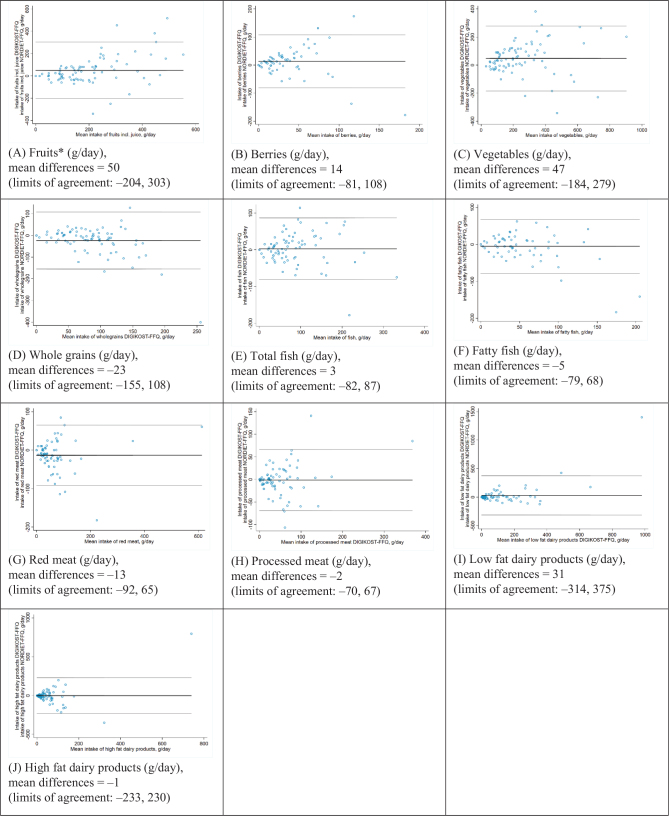
Bland–Altman plots, between DIGKOST-FFQ and NORDIET-FFQ, for intake of food groups grams per day, moderate and vigorous activity (minutes per day), height (centimeter), weight (kilograms). The black line represents the mean difference, and the gray lines represent the1.96 SDs limits of agreement, *n* = 81. *incl. berries and juice, maximum one glass of juice per day.

There was a significantly higher intake of ‘fruit’ by the DIGIKOST-FFQ compared to the NORDIET-FFQ, with a median difference of 29 g/day. The same was observed for ‘vegetables’ with a median difference of 36 g/day. Furthermore, a lower intake of ‘whole grains’ was observed with a median difference of –10 g/day (Table 4). Fig. 3A–D show the Bland-Altman analyses, with mean differences ranging from 14–50 g/day for ‘fruit’, ‘berries’, and ‘vegetables’ to –23 g/day for ‘whole grain’. A tendency of increasing differences with increasing intakes especially for ‘fruit’ and ‘berries’ was observed and a decrease in differences with increasing intakes for ‘whole grains’. However, the majority were within the 95% limits if agreement. Limits of agreements for fruit (–204, 303 g/day), berries (–81, 108 g/day), vegetables (–184, 279 g/day), and whole grains (–155, 108 g/day) were wide, and above the median of the two administrations (Table 4); however, the limits of agreements were more narrow for fruit- and vegetables intakes up to 250 g/day.

### Fish, meat, and dairy products

There were no significant differences observed between estimates of fish between DIGKOST-FFQ and NORDIET-FFQ, with a median difference of –1 and –3 g/day for ‘total fish’ and ‘fatty fish’, respectively. However, the DIGIKOST-FFQ did estimate a significantly lower intake for ‘red meat’, with a median difference of –11 g/day compared to NORDIET-FFQ. No significant differences were observed for ‘processed meat’ between the two methods. No significant differences were observed for ‘low or high fat dairy products’ (Table 4). Figure 3E–J shows the Bland-Altman analyses for ‘fish’, meat, and dairy products. Small differences in the mean were observed for these food groups except for ‘low fat dairy products’, with a mean difference of 31 g/day. Also a few outliers were observed for ‘red meat’ and ‘processed meat’ with intakes higher than 200 g/day, and for ‘low fat dairy products’ and ‘high fat dairy products’ for intakes higher than 400 g/day. A tendency of increasing difference with increasing means was observed causing wide limits of agreements. We observed an even distribution of differences above and below the mean difference.

## Discussion

We investigated the reproducibility and comparison of the new digital questionnaire, DIGIKOST-FFQ. Our study showed that the DIGIKOST-FFQ was able to reproduce reported intake of foods and other lifestyle factors according to the national recommendations on a group level. Furthermore, compared to the NORDIET-FFQ the DIGIKOST-FFQ showed improved estimates, for ‘fruits’, ‘vegetables’, ‘whole grain’, as ‘fruits’, ‘vegetables’ had been found to be underestimated and ‘whole grain’ overestimated by the NORDIET-FFQ (7).

### Reproducibility study

The present results indicate that the DIGIKOST-FFQ is able to reproduce lifestyle factors and dietary intake at the group level. Some differences in the dietary and lifestyle measures between the two administrations of the DIGIKOST-FFQ were observed; however, these differences were small compared to the average daily intake. Furthermore, based on the correlations and agreement classification, the DIGIKOST-FFQ was able to reproduce the reported dietary intake and lifestyle factors, that is shown by the correlation coefficients (≥ 0.6). This is also in accordance with a previous reproducibility study of a full web-based FFQ (20). Furthermore, analysis by the Bland-Altman plots, generally showed an increase in differences with higher reported intake, widening the plot causing a funnel-shape. The increased differences became more common when the intake was above the dietary recommendation (Norwegian FBDG) for fruit and vegetables > 250 g/day and whole grain >70/90 g/day for women and men, respectively. This indicated that the DIGIKOST-FFQ was able to measure whether individuals did not fulfill the Norwegian FBDG. Compared to a previous reproducibility study of a web-based FFQ (20), our limits of agreement for vegetables, red meat, and total fish were more narrow. On an individual level, there was a variation in measures between the two administrations, causing wider limits of agreements.

Other studies have observed a tendency of lower estimates of dietary intake at the second completion as compared to the first (20), leading to speculation of a learning effect. However, we did not see this trend, except for water, fruits, and vegetables, which might be explained by the weather, as it was extremely hot, for Norwegian standards, at the first completion. However, generally the observed differences were small and within the median average portion size of the food groups.

### Comparison study

The DIGIKOST-FFQ estimated higher intakes of ‘fruit and berries’, ‘berries’, and ‘vegetables’ and estimated lower intake of ‘whole grains’, compared to the reference method, NORDIET-FFQ. This is in accordance with the adjustments in DIGIKOST-FFQ based on the results from the validation of the NORDIET-FFQ, in which intakes of ‘fruit’, ‘berries’ and ‘vegetables’ were underestimated whereas ‘whole grains’ were overestimated (7). Thus, our results indicate constructive effects of the adjustments made in the DIGIKOST-FFQ. The DIGIKOST-FFQ estimated a lower intake of ‘red meat’ compared to the NORDDIET-FFQ, but with a satisfactory correlation. Furthermore, there were no significant differences between the dairy products and ‘total fish’ or ‘processed meat’, indicating that the measures by the NORDIET-FFQ and the DIGIKOST-FFQ worked equally well. The analysis by Bland-Altman plots in the comparison study, showed a tendency of a larger variability and uncertainty with a higher means of intake, giving funnel-shaped Bland-Altman plots, causing wide limits of agreements.

### Strengths and limitations

The participation rate in the present studies were lower than expected, with 350 participants versus the estimated 500 in the reproducibility study and the 81 participants versus the estimated 100 in the comparison study. This was not in line with previous studies evaluating longer and more time consuming FFQs, which have shown higher participation rates (20). However, the level of participation in epidemiological studies in general, seems to have decreased (21–23). A general decrease in volunteerism in the western world is observed, and scientific studies may have become increasingly demanding for participants (21).

Another limitation is that only one-fourth of the participants in the two studies were men, and a higher proportion of the participants in the present study had a high level of education as compared to the general Norwegian population (24). Yet, this selection is not expected to be an issue for the present results as the individuals serve as their own control. However, caution should be taken when generalizing the results to other individuals than to highly educated women. Lastly, in the comparison study we were unable able to evaluate tobacco use, sedentary time, and sleep as these factors were not included in the NORDIET-FFQ.

A strength of the two studies was the fact that a gap of 1–2 months between the competition of the two rounds of questionnaires helped us to investigate the reproducibility of DIGKOST-FFQ and do the comparison of the DIGKOST-FFQ and NORDIET-FFQ. A too long interval might result in actual change causing a low reproducibility and comparison results and a too short interval between completions might result in a learning effect, causing higher correlations (25).

Another strength of the comparison study is that the test method (DIGIKOST-FFQ) was completed before the reference method (NORDIET-FFQ), minimizing the learning effect from the reference method.

## Conclusion

The DIGIKOST-FFQ was able to reproduce reported dietary intakes, lifestyle- and demographic factors on a group level. Knowing the agreement between the two FFQs and the reproducibility of the DIGIKOST-FFQ is important for epidemiological studies, where the dietary intake and lifestyle assessments will be related to a variety of chronic disease outcomes. Furthermore, it was shown that the adjustments done in the DIGIKOST-FFQ based on the validation of the NORDIET-FFQ worked as intended.

## References

[1] Cade JE. Measuring diet in the 21st century: use of new technologies. Proc Nutr Soc 2017; 76(3): 276–82. doi: 10.1017/S0029665116002883 27976605

[2] Rusin M, Arsand E, Hartvigsen G. Functionalities and input methods for recording food intake: a systematic review. Int J Med Inform 2013; 82(8): 653–64. doi: 10.1016/j.ijmedinf.2013.01.007 23415822

[3] Amoutzopoulos B, Steer T, Roberts C, Cade JE, Boushey CJ, Collins CE, et al. Traditional methods v. new technologies – dilemmas for dietary assessment in large-scale nutrition surveys and studies: a report following an international panel discussion at the 9th International Conference on Diet and Activity Methods (ICDAM9), Brisbane, 3 September 2015. J Nutr Sci 2018; 7: e11. doi: 10.1017/jns.2018.4 29686860 PMC5906559

[4] Henriksen HB, Knudsen MD, Carlsen MH, Hjartaker A, Blomhoff R. A Short Digital Food Frequency Questionnaire (DIGIKOST-FFQ) assessing dietary intake and other lifestyle factors among Norwegians: Qualitative evaluation with focus group interviews and usability testing. JMIR Form Res 2022; 6(11): e35933. doi: 10.2196/35933 36346647 PMC9682459

[5] The Norwegian Directory of Health. Kostråd for å Fremme Folkehelsen og Forebygge Kroniske Sykdommer Metodologi og Vitenskapelig Kunnskapsgrunnlag: Nasjonalt Råd for Ernæring 2011. Oslo: Helsedirektoratet; 2011.

[6] Henriksen HB, Knudsen MD, Hjartåker A, Blomhoff R, Carlsen MH. Digital Food Frequency Questionnaire Assessing Adherence to the Norwegian Food-Based Dietary Guidelines and Other National Lifestyle Recommendations: Instrument Validation Study. J Med Internet Res. 2024 Apr 30;26:e53442. doi: 10.2196/53442. PMID: 38687986; PMCID: . 38687986 PMC11094607

[7] Henriksen HB, Carlsen MH, Paur I, Berntsen S, Bohn SK, Skjetne AJ, et al. Relative validity of a short food frequency questionnaire assessing adherence to the Norwegian dietary guidelines among colorectal cancer patients. Food Nutr Res 2018; 62: 1306. doi: 10.29219/fnr.v62.1306 PMC5846207 29545734

[8] Willett W, Lwnart E. Reproducibility and validity of food frequency questionnaires. In: Willett W, ed. Nutritional epidemiology. 3rd ed. Oxford Scholarship; New York: Oxford University Press; 2013, p. 96.

[9] Willett W. Correction for the effects of measurement error. In: Willett W, ed. Nutritional epidemiology. 3r ed. Oxford Scholarship 2013, New York: Oxford University Press; p. 287.

[10] Henriksen HB, Raeder H, Bohn SK, Paur I, Kvaerner AS, Billington SA, et al. The Norwegian dietary guidelines and colorectal cancer survival (CRC-NORDIET) study: a food-based multicentre randomized controlled trial. BMC Cancer 2017; 17(1): 83. doi: 10.1186/s12885-017-3072-4 28137255 PMC5282711

[11] Horn J, Kolberg M, Rangul V, Magnussen EB, Asvold BO, Henriksen HB, et al. Feasibility of a postpartum web- and phone-based lifestyle program for women with a history of preeclampsia or gestational diabetes: a pilot intervention study. Womens Health Rep (New Rochelle) 2023; 4(1): 345–57. doi: 10.1089/whr.2023.0039 37485436 PMC10357112

[12] Svendsen K, Henriksen HB, Ostengen B, Jacobs DR, Jr., Telle-Hansen VH, Carlsen MH, et al. Evaluation of a short Food Frequency Questionnaire to assess cardiovascular disease-related diet and lifestyle factors. Food Nutr Res 2018; 62: 1370. doi: 10.29219/fnr.v62.1370 PMC5917418 29720928

[13] Svendsen K, Telle-Hansen VH, Morch-Reiersen LT, Garstad KW, Thyholt K, Granlund L, et al. A randomized controlled trial in Norwegian pharmacies on effects of risk alert and advice in people with elevated cardiovascular risk. Prev Med Rep 2018; 12: 79–86. doi: 10.1016/j.pmedr.2018.08.004 30191097 PMC6125803

[14] Henriksen HB, Berntsen S, Paur I, Zucknick M, Skjetne AJ, Bohn SK, et al. Validation of two short questionnaires assessing physical activity in colorectal cancer patients. BMC Sports Sci Med Rehabil 2018; 10: 8. doi: 10.1186/s13102-018-0096-2 29854408 PMC5975662

[15] Cade JE, Burley VJ, Warm DL, Thompson RL, Margetts BM. Food-frequency questionnaires: a review of their design, validation and utilisation. Nutr Res Rev 2004; 17(1): 5–22. doi: 10.1079/NRR200370 19079912

[16] Margetts BM, Nelson M. Design concepts in nutritional epidemiology. New York: Oxford University Press; 1997.

[17] Hankin JH, Wilkens LR, Kolonel LN, Yoshizawa CN. Validation of a quantitative diet history method in Hawaii. Am J Epidemiol 1991; 133(6): 616–28. doi: 10.1093/oxfordjournals.aje.a115934 2006649

[18] Bland JM, Altman DG. Statistical methods for assessing agreement between two methods of clinical measurement. Lancet 1986; 1(8476): 307–10. doi: 10.1016/S0140-6736(86)90837-8 2868172

[19] Schmidt ME, Steindorf K. Statistical methods for the validation of questionnaires – discrepancy between theory and practice. Methods Inf Med 2006; 45(4): 409–13. doi: 10.1055/s-0038-1634096 16964357

[20] Carlsen MH, Andersen LF, Hjartaker A. Reproducibility and feasibility of an online self-administered food frequency questionnaire for use among adult Norwegians. Food Nutr Res 2021; 65: 7561. doi: 10.29219/fnr.v65.7561 PMC8634621 34908922

[21] Galea S, Tracy M. Participation rates in epidemiologic studies. Ann Epidemiol 2007; 17(9): 643–53. doi: 10.1016/j.annepidem.2007.03.013 17553702

[22] Halbesleben JR, Whitman MV. Evaluating survey quality in health services research: a decision framework for assessing nonresponse bias. Health Serv Res 2013; 48(3): 913–30. doi: 10.1111/1475-6773.12002 23046097 PMC3681236

[23] Keeble C, Baxter PD, Barber S, Law GR. Participation rates in epidemiology studies and surveys: a review 2007–2015. Internet J Epidemiol 2016; 14: 1–14.

[24] Norway S. Educational attainment of the population 2022. Available from: https://www.ssb.no/en/utdanning/utdanningsniva/statistikk/befolkningens-utdanningsniva [Cited: 24 August 2022]

[25] Cade J, Thompson R, Burley V, Warm D. Development, validation and utilisation of food-frequency questionnaires – a review. Public Health Nutr 2002; 5(4): 567–87. doi: 10.1079/PHN2001318 12186666

